# Dermatobia Noxialis

**Published:** 1911-01-21

**Authors:** 


### DERMATOBIA NOXIALIS.

Amongst emigrants returning from foreign countries various unusual parasitic affections are relatively common, and in connection with these may be mentioned the *Dermatobia noxialis*.

In a recent case an infant, aged ten months, lately returned from South America, had been left sleeping on the ground a month previously, and when seen it had three round, soft, elastic swellings, each the size of a walnut, on the scalp. These swellings were not reddened, but displayed a central small opening, from which a frothy, whitish, thread-like serum exuded. From each swelling was ultimately extracted a whitish, globular segmented worm, the larva of a fly called *Dermatobia noxialis*. The swellings then rapidly subsided.

This fly abounds in tropical America, and attacks monkeys, dogs, and cattle as well as men. Humboldt relates having seen Indians whose abdomens were absolutely covered with the small tumours they excite. The eggs or larvæ are deposited in the skin by the adult fly by means of a special mechanism, but it has also been proved that the larvæ hatched from eggs on the leaves of trees attach themselves to the skin of man or beast and penetrate into the subcutaneous tissue. Once recognised, the condition can be readily cured by a simple surgical measure; if, however, a little cutting operation is objected to, the larvæ can be killed by means of a cotton pad soaked in a 4 per cent. solution of carbolic acid and then extracted. The chief difficulty may be in recognising the nature of the lesion when it is seen for the first time.

